# Online Attitudes and Information-Seeking Behavior on Autism, Asperger Syndrome, and Greta Thunberg

**DOI:** 10.3390/ijerph18094981

**Published:** 2021-05-07

**Authors:** Ingjerd Skafle, Elia Gabarron, Anders Dechsling, Anders Nordahl-Hansen

**Affiliations:** 1 Faculty of Health and Welfare, Østfold University College, 1671 Kråkerøy, Norway; 2 Faculty of Education, Østfold University College, 1757 Halden, Norway; elia.gabarron@ehealthresearch.no (E.G.); anders.dechsling@hiof.no (A.D.); anders.nordahl-hansen@hiof.no (A.N.-H.); 3 Norwegian Centre for E-Health Research, 9038 Tromsø, Norway

**Keywords:** autism spectrum disorders, Asperger syndrome, social media, Twitter messaging, Google Trends, public health, sentiment analysis, content analysis

## Abstract

The purpose of this study was to examine Internet trends data and sentiment in tweets mentioning autism, Asperger syndrome, and Greta Thunberg during 2019. We used mixed methods in analyzing sentiment and attitudes in viral tweets and collected 1074 viral tweets on autism that were published in 2019 (tweets that got more than 100 likes). The sample from Twitter was compared with search patterns on Google. In 2019, Asperger syndrome was closely connected to Greta Thunberg, as of the tweets specifically mentioning Asperger (from the total sample of viral tweets mentioning autism), 83% also mentioned Thunberg. In the sample of tweets about Thunberg, the positive sentiment expressed that Greta Thunberg was a role model, whereas the tweets that expressed the most negativity used her diagnosis against her and could be considered as cyberbullying. The Google Trends data also showed that Thunberg was closely connected to search patterns on Asperger syndrome in 2019. The study showed that being open about health information while being an active participant in controversial debates might be used against you but also help break stigmas and stereotypes.

## 1. Introduction

Autism spectrum disorder (autism from hereon) has become part of popular culture [1]. Films and TV series [2] with fictional autistic characters are highly popular [3]. Traditional media, such as newspapers, TV, and radio, cover autism-related content regularly [4]. Internet and social media are no exceptions. With 4.66 billion active Internet users worldwide and close to 4.14 billion active users on social media as of October 2020 [5], online social media have an enormous outreach and offer opportunities for individuals to voice unfiltered opinions, including autism information. Internet and social media platforms have become common sources of information and provide open and accessible arenas for communication. Social media and search engines also give vital indications of the general public’s agenda and of what might be “trending” at any given time.

In August 2018, newspapers in Scandinavia printed stories about a young girl, Greta Thunberg, who skipped school to protest against the neglect and denial of climate change and global warming [6,7]. Beginning in fall 2018 and continuing throughout the year 2019, worldwide school strikes for climate were being arranged due to her commitment. Thunberg reached global fame in September 2019, as she spoke to the 2019 United Nations Climate Action Summit where she gave a famous speech about the dangers of global warming. She was named “person of the year” by Times Magazine in December 2019 [8]. In interviews, Thunberg herself has been open about being diagnosed with Asperger syndrome, a diagnosis that became part of the umbrella diagnosis Autism spectrum disorder in the Diagnostic and Statistical Manual of Mental Disorders 5 [9]. Asperger syndrome (Asperger from hereon) is characterized by difficulties with social interactions and a restricted repertoire of interests and activities. There is no general delay in language or cognitive development [10]. Thunberg herself has referred to Asperger as her “superpower” on Twitter [11].

Research on Twitter content related to mental health conditions and disorders, such as schizophrenia, psychosis, and depression, has been undertaken before [12,13], as well as analyses of the sentiment expressed on diabetes [14]. Some studies have investigated Twitter use related to autism. Zhang and Ahmed [15] compared information sharing of over 379 health conditions on Twitter to uncover trends and patterns of online user activities. They collected 1.5 million tweets generated by 450,000 Twitter users. The results showed that autism was among the top five communities and one of the health conditions with the largest network and interest on Twitter. In a recent study, patterns and themes of tweet content, the sentiment within tweets, and intercommunications between stakeholders within autism communities were examined. The results indicated that the Twitter content was primarily related to empowerment and support [16]. Beykikhoshk et al. [17] explored how Twitter could be used as a data-mining source to learn about the behavior, concerns, and needs of the population affected by autism. The most important finding concerned the nature of the topics arising in the autism subset of tweets and the fact that the tweets in this subset were very rich in information, which again has a potentially high value for public health officials and policymakers. Tomeny et al. [18] examined temporal trends, geographic distribution, and demographic correlates of autism-related anti-vaccine beliefs on Twitter from 2009 to 2015. Their results indicated that the volume of autism-related anti-vaccine beliefs online was alarming and that anti-vaccine tweets coincided with news events and tended to cluster geographically. There is also an evolving area of research on information-seeking behavior. Tárraga-Minguez et al. [19] analyzed Google search peaks for the terms “autism” and “Asperger” in Spain, from 2015 to 2019. The authors concluded that social marketing campaigns played an important role in the normalization of autism, and also that the way autism was portrayed in news media and on social media networks played a powerful role in shaping the perception of autism. In addition, the authors found a strong link between the increased volume of searches for the term “Asperger” and major news events regarding Greta Thunberg.

A recent study [20] examined help-seeking behavior in connection with Thunberg’s increased media attention. Results showed that in search interest on Google, there was a strong association between the terms Asperger syndrome and Greta Thunberg, as these two terms peaked approximately at the same time during 2019. The study also suggested a connection between help-seeking behavior and Thunberg’s exposure, as they observed a steadily increased Internet traffic for the Asperger/Autism Network and Autism Speaks websites from June to December 2019. Moreover, the study suggested that Thunberg’s media coverage may have had a positive impact in reducing stigma around Asperger.

### Aim

In this study, we examined attitudes and sentiment in tweets and Internet search trends mentioning autism, Asperger syndrome, and Greta Thunberg, during 2019. The study had two objectives: (1) to examine and compare sentiment and attitudes in viral tweets mentioning autism and tweets mentioning autism in combination with Asperger and/or Thunberg and (2) to investigate Internet users’ information-seeking behavior regarding autism, Asperger syndrome, and Thunberg, both during 2019.

## 2. Materials and Methods

This study had a mixed-method design. We used a cross-sectional analysis of data generated from Twitter and Google Trends. The study design was inspired by a study conducted by Gabarron et al. [14], where they used sentiment analysis of online attitudes towards diabetes, and a more recent study of Twitter usage about autism, where qualitative content analysis was used to analyze tweets [16].

### 2.1. Data Extraction

#### 2.1.1. Twitter Data

Twitter is one of the most popular social media platforms and has 187 million monetizable daily active users [21]. Tweets are limited to 280 characters and may include up to four pictures, a GIF, or a video [22]. We searched for all viral tweets on autism that were published every month in 2019 (here we considered a tweet to be viral when it got more than 100 likes). We used the advanced search engine from Twitter and searched for tweets with more than 100 likes that included: autism OR autistic OR aspie OR Asperger OR ASD. Our search was limited to the English language. All identified posts were extracted. We extracted tweet contents and their number of likes, comments, and shares. No personal data from the Twitter accounts were extracted. Major demographic characteristics (age, gender, race/ethnicity, and socioeconomic status) were not reported, as our main focus was on attitudes and sentiment expressed in the tweets. We did not include any direct quotations of tweets to protect the Twitter users’ anonymity.

#### 2.1.2. Google Trends Data

Google is the world’s leading search engine tool and had a market share of 86.8% in July 2020 [23]. Google Trends normalizes traffic data concerning searches on a scale from 0 (<1% of the peak volume) to 100 (peak of traffic). We used Google Trends to gather the volume of search queries carried out in Google Search during 2019 about autism, Asperger syndrome, Autism spectrum disorder, and Greta Thunberg to assess public interest in these topics.

### 2.2. Data Analysis

#### 2.2.1. Content Analysis

Content analysis is a systematic coding and categorizing approach used to explore a large amount of textual information to establish the trends and patterns of words used, their frequencies, relationships, and structures [24]. Precisely because it makes it possible to analyze data qualitatively and, at the same time, to quantify them, we chose content analysis over thematic analysis, as their main difference lies in the possibility of measuring the frequency of different categories in content analysis [25]. In addition, when exploring data in content analysis, one uses categories instead of abstract themes [25], and we found this approach to be most useful in this study. In inductive content analysis, the categories are derived from the data. If there is not enough knowledge about the phenomenon or if this knowledge is fragmented, the inductive approach is recommended [26].

Content analysis uses a descriptive approach in both coding of the data and the interpretation of quantitative counts of the codes. The basic coding process in content analysis is to organize large quantities of text into much fewer content categories [27]. Within our database, we identified posts referring specifically to Asperger (those that included any of the terms “Asperger” or “Aspi”) and tweets referring to Greta Thunberg (those that included the terms “Greta, “Thunberg” and/or “Swed*”). Two authors (I.S. and A.D.) categorized the tweets based on their content and solved any disagreement through discussions. The tweets were then designated into categories of action (defending, praising, and stigmatizing attitudes). 

#### 2.2.2. Sentiment Analysis

The sentiment analysis of the tweets was carried out with AFiNN [28]. AFiNN is a lexicon or wordlist consisting of 2477 words that can analyze the language used in microblogs such as Twitter. AFiNN uses a scoring range where each word scores from −5 (very negative) to +5 (very positive) [28]. Words below a score of −0.05 are tagged as negative and words above 0.05 as positive. Words in between are considered neutral [29]. Statistical analyses were performed with SPSS (version 25; IBM Corp.: Armonk, NY, USA).

## 3. Results

### 3.1. Sentiment Analysis

A total of 1074 viral tweets (>100 likes) mentioning autism, Asperger, and/or ASD were posted in 2019. These viral tweets were extracted, and the sentiment analysis of the whole sample showed a neutral sentiment (−0.23), leaning slightly towards a negative sentiment. Among these, 49 tweets (4.6%) specifically mentioning “Asperger” or “Aspi*” had a significantly more negative sentiment than the tweets that did not include those words (−1.94 vs. −0.14, *p* = 0.008). Tweets specifically mentioning “Greta, “Thunberg”, and/or “Swed*” also had a more negative sentiment than the tweets that did not include these words (−1.28 vs. −0.17, *p* = 0.090). The sentiment analysis of the whole sample is reported in Table 1.

**Figure 1:**
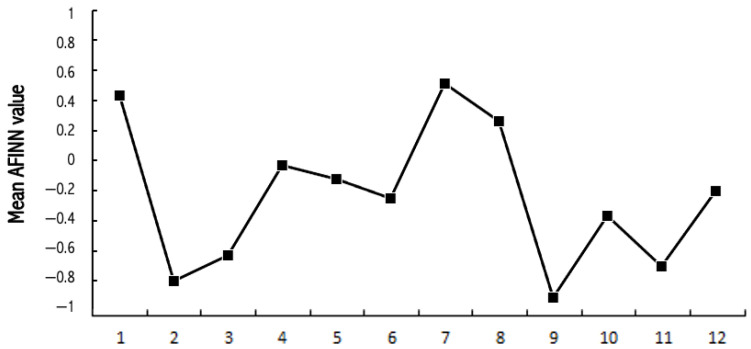
Graph showing the mean of the sentiment of the whole sample of tweets for each month of 2019.

The sentiment of the whole sample of tweets showed a monthly variation of negative and positive values, with a clear spike on the positive note in January, a negative spike in February, jumping to a positive spike in July, and then a clear spike towards negative sentiment in September (see Figure 1).

**Figure 2:**
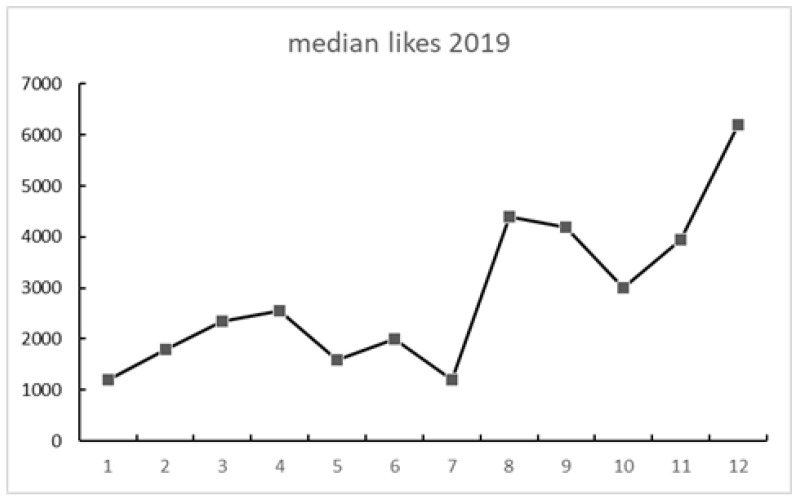
Median monthly likes of the total sample during 2019.

**Figure 3:**
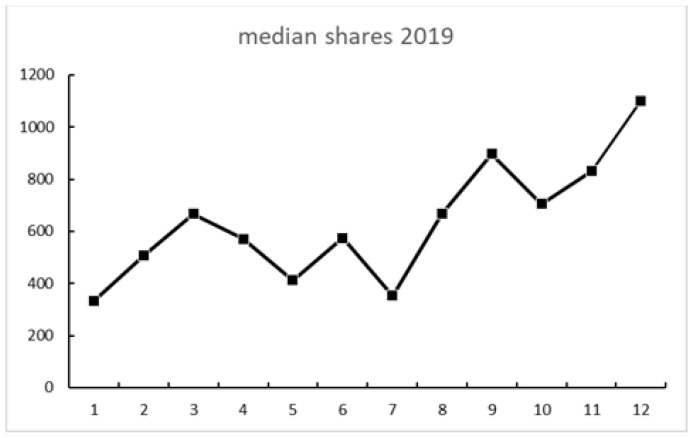
Median monthly shares of the total sample during 2019.

**Figure 4:**
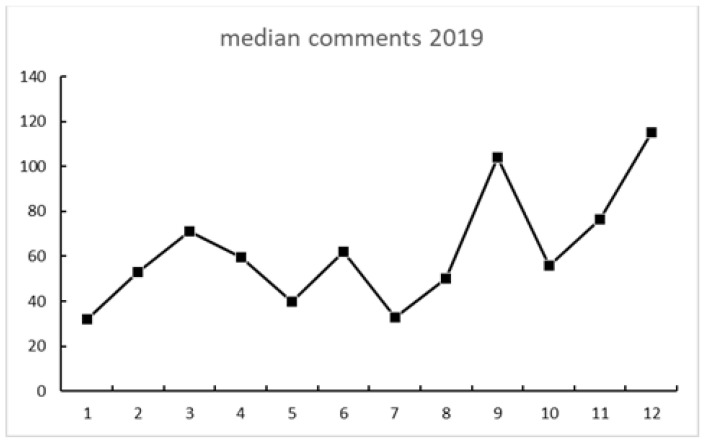
Median monthly comments of the total sample during 2019.

Figure 2, Figure 3 and Figure 4 show the engagement in the total sample of tweets. All figures show a somewhat similar trend: activity rose at some point in August and September, then went down in October, and then spiked in December. There were less shares than likes and even fewer comments (see Figure 4).

### 3.2. Content Analysis

**Figure 5:**
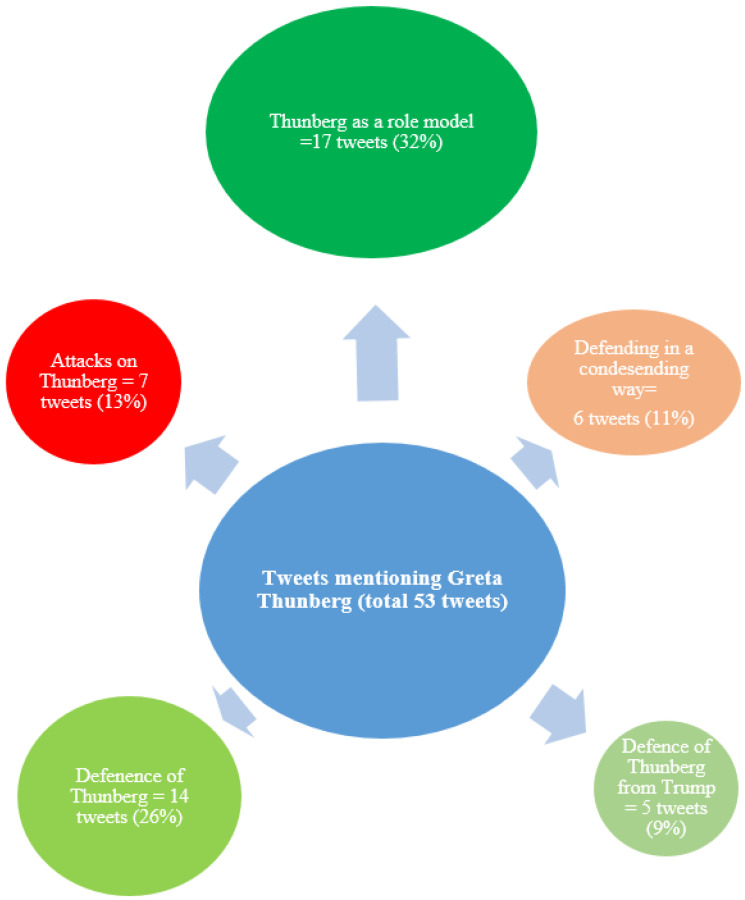
The Five Largest Categories of Tweets Mentioning Greta Thunberg. This figure illustrates the five largest categories of tweets mentioning Greta Thunberg, leaving out the category “Unable to understand tweet” (1 tweet). The colors in green tones indicate a positive attitude/sentiment, whereas the red tones a negative attitude/sentiment.

The content analysis showed that the tweets mentioning autism in combination with specifically Greta Thunberg had an overall positive attitude towards her and that she was seen as a role model. Many Twitter users defended her from various attacks. Some tweets were negative towards her and either attacked her because of her diagnosis or defended her in a condescending way (Figure 5). Even though the majority of the tweets were on Thunberg’s side, the tweets that attacked her stirred more engagement in terms of likes, shares, and comments (Table 2).

**Figure 6:**
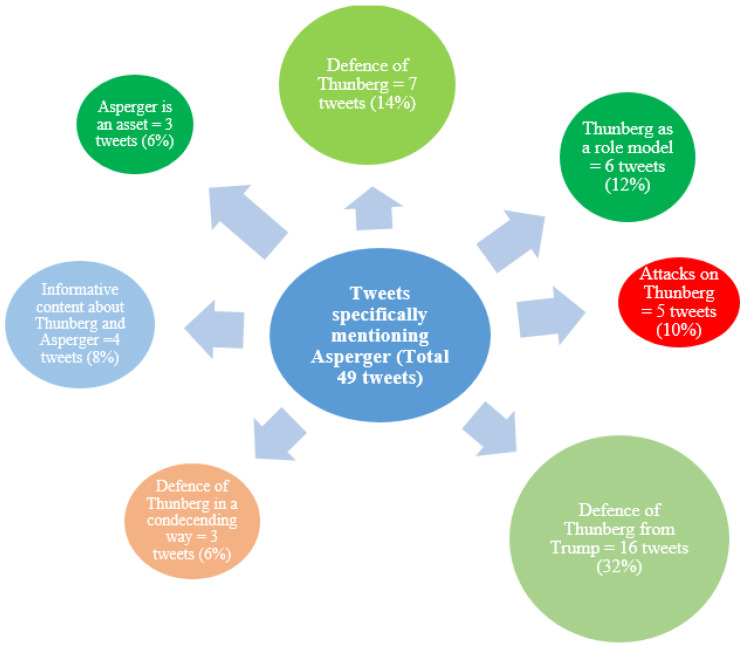
The Seven Largest Categories of Tweets Mentioning Asperger. This figure illustrates the seven largest categories of tweets specifically mentioning Asperger, leaving out the categories “Asperger is not an excuse” (1 tweet), “Informative about Asperger” (1 tweet), “Reaching out to a celebrity” (1 tweet), and “Unable to understand tweet” (2 tweets). The colors in green tones indicate a positive attitude/sentiment whereas the red tones a negative attitude/sentiment. Blue means neutral.

The content analysis showed that 83% of the tweets with content related to autism in combination with Asperger also had content about Thunberg (Figure 6). The largest category included tweets (32%) that defended Thunberg from the then sitting President Donald Trump. The category of tweets that stirred the most engagement in terms of likes was the one where Thunberg was defended from attacks using Asperger against her (14%). However, the tweets that attacked Thunberg or condescendingly defended her, despite being relatively rare, stirred quite a lot of engagement in terms of likes, shares, and comments (Table 3).

### 3.3. Google Trends

**Figure 7:**
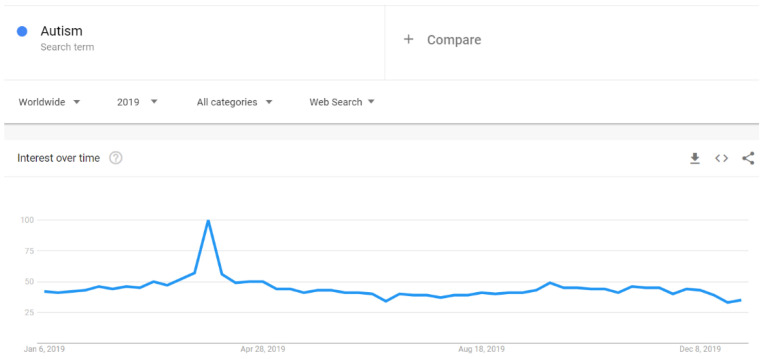
Google search pattern on Autism.

**Figure 8:**
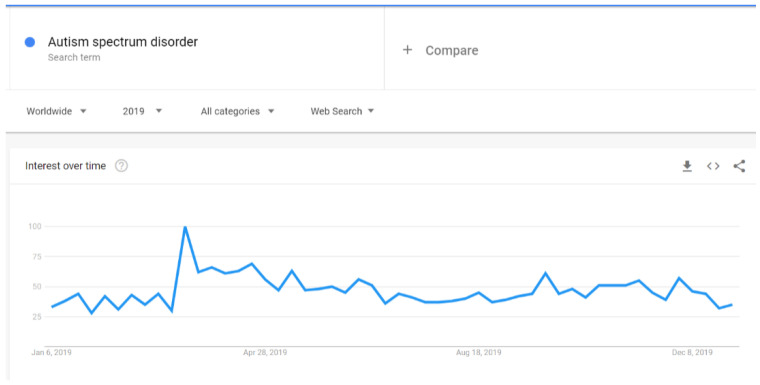
Google search pattern on Autism spectrum disorder.

**Figure 9:**
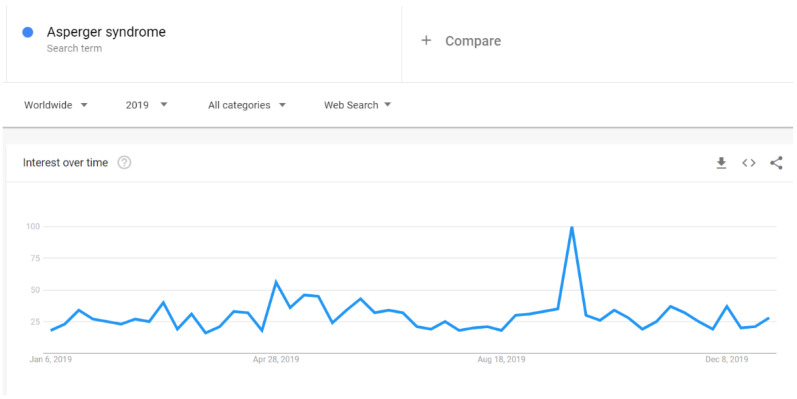
Google search pattern on Asperger syndrome.

**Figure 10:**
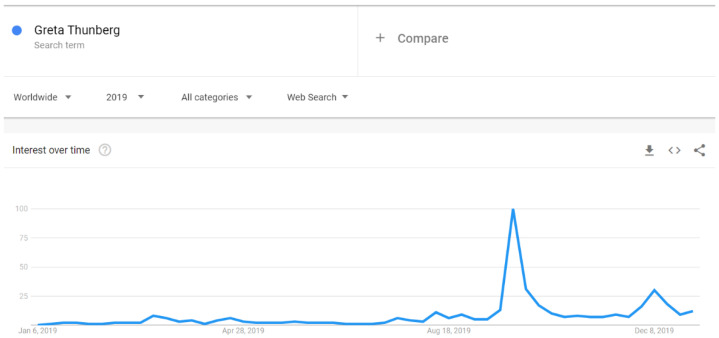
Google search pattern on Greta Thunberg.

There were increases in Google searches for the term “autism” in early April 2019 (Figure 7). Similarly, the search for “autism spectrum disorder” spiked in early April 2019, but not as clearly as for the word “autism”. The expression “autism spectrum disorder” had a noticeable increase also in early December 2019 (Figure 8). The search pattern for “Asperger syndrome” looked different; it had a clear spike in September 2019 (Figure 9). The search pattern for “Greta Thunberg” (Figure 10) was as the one of “Asperger Syndrome”, as both spiked in September 2019.

## 4. Discussion

In this study, we first examined and compared sentiment and attitudes in viral tweets mentioning autism and specifically mentioning Asperger and/or Greta Thunberg, in 2019. Subsequently, we compared Internet users’ information-seeking behavior regarding autism, Asperger syndrome, and Thunberg, during 2019. Our findings suggested that the overall sentiment on autism during 2019 in these viral tweets (*n* = 1074) was neutral. However, the sentiment showed great monthly variation: clear negative spikes in the AFiNN values in February and September and a positive spike in July. When comparing the numbers from the sentiment analysis of the total sample with the sentiment on Asperger and Thunberg, we found that the sentiment in the sample of tweets specifically mentioning Asperger was significantly more negative than the one in the total sample. The sample with tweets mentioning Thunberg also had a more negative sentiment than the total sample, but it was not a significant difference. To look closer into what the types of negativity (and positivity) these tweets expressed in the sentiment analysis, we performed a content analysis on the Asperger sample (53 tweets) and the Thunberg sample (49 tweets) because the sentiment analysis indicated a form of negativity not only towards Asperger but also towards Thunberg. The Twitter engagement might have suggested a form of negativity because these two categories were less liked and shared than the total sample. However, hitting the like button is a different process than commenting, and the Asperger and Thunberg categories both received the most comments. Commenting requires a process that is more cognitive and emotive, whereas liking is a response more similar to automatic processing [30]. Therefore, it is interesting to see that these categories could have caused engagement on a deeper level, due to a higher number of mean comments than the overall sample had. Furthermore, when we inspected the data through qualitative content analysis, the picture seemed even more complex. However, the numbers in the categories in the content analysis were too small and thus not generalizable entities.

### 4.1. Thunberg as a Controversial Role Model

The main findings of the content analysis were that of the tweets mentioning Thunberg, (1) the largest category (32%) included tweets that expressed that Thunberg was a role model—for example, because of saying that Asperger was her “superpower”—and (2) the second largest category (26%) included tweets that defended her from various attacks. Both categories can be labeled as positive towards Thunberg. There was one category (13%) that was negative towards her and contained attacks on her, where autism or Asperger was mentioned and used against her. This can be considered cyberbullying and could hurt people who regard Thunberg as a role model or have the same condition. Previous studies have shown that autistic children and adolescents are more vulnerable when it comes to traditional bullying [31,32]. Furthermore, recent studies have shown that autistic adolescents are also at risk of being victims of cyberbullying and that there is an association between cyberbullying victimization and depression and anxiety among teens with autism [33,34]. Nevertheless, the content analysis showed that a majority of the tweets specifically mentioning Thunberg were on “her side”. The sentiment analysis, however, picked up a somewhat negative attitude in the Thunberg sample of tweets.This can be seen in relation to two of the categories in the content analysis that consisted of tweets that either defended Thunberg in general or defended her against the, then sitting, American president, Donald Trump. The negativity the sentiment analysis picked up was therefore not necessarily aimed at Thunberg or Asperger syndrome (although it was that, too), but could also be due to a negative tone in the climate change debatewith more swearwords and name-calling.

The content analysis indicated that Thunberg was seen as an empowering figure, as she spoke openly about her Asperger diagnosis and said without shame that it was an asset and a superpower [11]. On the one hand, one would expect people to view persons with autism differently when seeing this very resourceful and determined young individual, regardless of their stance in the climate change debate. Thunberg may be a key figure in reducing the stigma surrounding Asperger and autism [20]. However, opponents in a polarized debate, such as the climate change controversy, might use whatever arguments they can find to discredit their foes. Another category in our content analysis, which also could be considered as negative towards Thunberg, although in a subtler way, was the one that contained tweets that condescendingly defended her (11%), meaning that they contained words that seemed kind but treated her as ignorant and the author of the Tweet as superior, calling her a child, unstable, autistic (in a negative manner), or mentally ill. These tweets contained the message that because of these characteristics, one should leave her alone and not take her seriously. Whether these tweets were written out of misinformed compassion or on purpose, to discredit her, is not known. The latter option is crueler and considered cyberbullying, but the former is perhaps more disheartening, as it shows the stigma and misinformation surrounding autism. Jung et al. [35] examined 1.7 million tweets related to Thunberg, and the results also showed, through sentiment analysis, that she was a controversial figure who stirred both positive and negative tweets. The authors also concluded that political polarization heavily influenced the analyzed tweets. In our study, we can see that the viral tweets that attacked Thunberg stirred the most engagement, as these tweets received, on average, more likes, comments, and shares. This confirmed that she was a controversial figure. The fact that her young age and Asperger diagnosis were used against her might not be surprising, but it is not reassuring in the context of open online debates whereencouraging young people to participate in politics, and also encouraging people with health conditions such as autism to get involved is essential. Seeing how Thunberg is treated might be discouraging. On the other hand, it might be encouraging to see how she keeps going despite criticism and that many people defend her online.

### 4.2. Close Association between Thunberg and Asperger in 2019

Another important finding was that when we extracted tweets that specifically mentioned Asperger (from the total sample of viral tweets mentioning autism), 83% of them also mentioned Thunberg. This indicated that in 2019, Thunberg (tweets about her) was in close association with Asperger. The largest category in this sample was the one that contained tweets that defended Thunberg from Trump (32%). Thunberg had become a focal point in the highly controversial debate of climate change, and she had also been given attention by Trump on Twitter. Many of the viral tweets included in the current sample stemmed from this attention that seemed to arise during and after Thunberg’s speech at the United Nations Climate Summit in September 2019. Around that time, in December 2019, she also became Time Magazine’s person of the year. On both occasions, Trump commented on Thunberg as a person. Although he did not address her diagnosis directly in these tweets, the comments from other users did.

A strong association between Internet searches on Asperger and Thunberg was also seen in Google Trends data. The search terms Greta Thunberg and Asperger syndrome (Figure 9 and Figure 10) both peaked in September 2019 and followed a quite similar search pattern. However, the search terms autism and autism spectrum disorder both peaked in April (Figure 7 and Figure 8) during the Autism Awareness month. This has been a trend in recent years and is supported by a study on the online search interest on autism [36]. Considering that Asperger syndrome is no longer part of DSM-5, one could also expect a similar rise in interest in searches about autism during the times when Thunberg got a lot of attention (i.e., September and December 2019). Not seeing the same association between autism spectrum disorder and Thunberg may be due to the general public’s lack of familiarity with the link between Asperger syndrome and autism spectrum disorder. On the other hand, there might be a stronger association than the one that Google Trends can pick up due to the limitations of this tool [37]. In our total sample of viral tweets (n = 1074), however, we could see a clear spike towards a negative sentiment in September (Figure 1). Similarly, the three graphs of median likes, shares, and comments (Figure 2, Figure 3 and Figure 4) show that around September and December 2019, these activities increased for the total sample. One might speculate whether this was due to Thunberg’s elevated media attention at that particular time. 

### 4.3. Limitations and Future Research

This study had several limitations. First, we only analyzed viral tweets, that is posts that got the most likes. We know that there are tweets that receive other types of online engagement, such as shares and comments (also seen in our study), that are equally important in the overall picture. Therefore, it would be interesting in future research to examine tweets with a different reach and engagement parameter. Moreover, not knowing the context in which these tweets were written is a limitation when it comes to interpreting the intended purpose behind the tweets. Furthermore, we did not investigate whether some of the tweets stemmed from “bots”, which are Twitter accounts that automate content [38]. These bots are created with different purposes, including malicious intentions [39]. It is not known how the public opinion can be manipulated by bots, nor how the possible presence of bots could have affected our conclusions [39,40]. Another limitation is that we only focused on messages posted in English. A majority of Twitter users (69.3 million) are from the US, but Japan, with 50.9 million users, is the second leading country when it comes to Twitter [21]. Future studies should therefore also look at tweets in other languages than English. Moreover, discourse on Twitter is typically dominated by a minority of tweeters, who are responsible for a majority of all tweets [41]. Thus, there is a large group of “silent” tweeters who never tweet or retweet. It would therefore be interesting to study the “silent majority” in future research.

There are some limitations in the tools we used in this study. AFiNN is not able to detect irony and sarcasm, so the sentiment analysis was not flawless [28]. Previous research has shown that tweets about Thunberg were strongly influenced by political polarization due to the climate change controversy [35]. Our study investigated tweets regarding Thunberg and autism. However, we do not know precisely how her role in the climate change controversy influenced the sentiment analysis in our study, but because she is primarily known for her environmental activism, it most likely played a key role. This was also confirmed in the content analysis. There are also limitations when it comes to utilizing Google Trends. It should not be considered as polling data and, according to Google, it should be considered as one data point among others before making any conclusions [37].

## 5. Conclusions

Although earlier studies have indicated that social media attention can be positive in raising awareness about autism, social media attention and user interactions are complex. Media attention and autism have been investigated in several ways in more traditional media outlets from newspapers [4,42], to film and TV [1,3]. Traditional media are more controlled in various ways, whereas social media are open to anyone making it easier to voice opinions. In this study, we found that social media, with the case example of Twitter, contributed to both positive and negative content concerning autism and Asperger. There are also clear indications that persons such as Thunberg raised attention to and interest in Asperger. When inspecting Google Trend data, we found that during 2019, Asperger was closely associated with her; we also found this association in the content analysis. On Twitter, she was subject to debates both in regard to her person and her opinions in the climate change debate, but her Asperger diagnosis also received a lot of attention and engagement. Thunberg was regarded as a role model in many ways. However, she was also subject to cyberbullying by people who either wanted to discredit her or had a stereotypically negative view of people with an autism diagnosis.

It is important that future research investigates the mechanisms of how social media influence the general public’s opinion about autism spectrum disorder, and it is equally important to understand how it might affect persons with the diagnosis and their families, as social media probably will play a key role in future information campaigns about autism.

## Figures and Tables

**Table 1:** Average sentiment of the tweets and number of likes, shares, and comments.

Sentiment Analysis	AFiNN Value Mean	Likes Mean	Shares Mean	Comments Mean
All viral tweets mentioning (“autism” or “#autism”) OR (“Asperger” or “#Asperger”) OR (“ASD” or “#ASD”) (n = 1074)	−0.23	9577.85	2284.48	239.48
Tweets specifically mentioning Asperger				
Yes (n = 49; 4.6%)	−1.94	7187.71	1472.29	375.69
No (n = 1025; 95.4%)	−0.14	9692.11	2323.31	232.97
Tweets specifically mentioning Greta Thunberg				
Yes (n = 53; 4.9%)	−1.28	6542.74	1511.58	328.34
No (n = 1021; 95.1%)	−0.17	9735.40	2324.61	234.87

**Table 2:** Engagement with tweets about Greta Thunberg.

Categories	Definition of Categories	N	Likes Mean (CI 95%)	Shares Mean (CI 95%)	Comments Mean (CI 95%)
Thunberg as a role model	Thunberg being a role model who uses autism/Asperger syndrome as a superpower/gift	17 (32%)	6508.00 (−969.07–13,985.07)	1168.00 (−98.03–2434.03)	78.76 (15.92–141.61)
Defense of Thunberg	Defending Thunberg against attacks on her in media or social media	14 (26%)	4641.36 (2194.12–7088.59)	1391.64 (608.99–2174.29)	281.64 (72.01–491.28)
Attack on Thunberg	Tweets that are very negative towards Thunberg and that attack her in several ways and often use autism or Asperger against her	7 (13%)	11,296.29 (−5195.84–27,788.41)	3036.71 (−1412.50–7485.93)	864.86 (−329.76–2059.48)
Defending Thunberg in a condescending way	Tweets that ask people to leave Thunberg alone because she is autistic, terrified, vulnerable, a child, etc.	6 (11%)	5600.00 (2690.96–8509.04)	1231.33 (476.00–1986.66)	364.50 (128.39–600.61)
Defending Thunberg from President Trump	Tweets that explicitly defend Thunberg from tweets posted by the, then incumbent, President Trump, where he, for example, asked her to work on her anger management problem	5 (9%)	4920.00 (987.24–8852.76)	1057.60 (295.55–1819.65)	357.60 (−108.13–823.33)
Information about Thunberg and autism	Informative tweets about facts regarding Thunberg and autism diagnosis	3 (5%)	11,233.33 (−24,863.11–47,329.77)	2280.67 (−4646.20–9207.53)	695.33 (−1482.01–2872.68)
Unable to understand the message	Tweets that were hard to understand or did not make sense	1 (1%)	176.00 (.)	0.00 (.)	5.00 (.)
Total		53 (100%)	6542.74 (3494.44–9591.03)	1511.58 (845.73–2177.44)	328.34 (166.44–490.24)
*p*-value			0.437	0.201	0.008

Kruskal–Wallis test.

**Table 3:** Engagement with tweets on Asperger.

Categories	Definition of Categories	N	Likes Mean (CI 95%)	Shares Mean (CI 95%)	Comments Mean (CI 95%)
Defending Thunberg against Trump	Tweets that explicitly defends Thunberg from tweets posted by the, at that time incumbent, President Trump in which he mocks Thunberg	16 (32%)	8212.50 (2239.56–14,185.44)	1795.00 (689.51–2900.49)	679.25 (166.79–1191.71)
Defending Thunberg from attacks where Asperger is used against her	Defending Thunberg against attacks in media or on social media where Asperger is used to discredit her. Links to other Twitter users or online news articles.	7 (14%)	12,600.00 (1497.23–23,702.77)	1311.00 (64.72–4413.28)	249.00 (25.83–472.17)
Thunberg as a role model	Tweets that praise Thunberg for promoting that Asperger diagnosis is a gift and a superpower. She is a role model for the autistic community and a role model in general.	6 (12%)	4700.00 (1533.56–7866.44)	819.33 (463.81–1174.85)	75.17 (29.16–121.18)
Attacks on Thunberg using Asperger against her	Tweets that discredit Thunberg as a serious person in the climate debate due to her Asperger diagnosis	5 (10%)	4694.20 (−2952.60–12,341.00)	1311.00 (−891.34–3513.34)	245.20 (−1.32–491.72)
Informative content about Thunberg and Asperger	Neutral and informative content about Asperger and Thunberg	4 (8%)	8479.50 (−12,334.32–29,293.32)	1715.00 (−2330.43–5760.43)	524.75 (−736.80–1786.30)
Defending Thunberg in a condescending way	Defending Thunberg, but in a condescending way. Words such as child, autistic, Asperger, vulnerable, mentally ill are used.	3 (6%)	6300.00 (−2728.74–15,328.74)	1432.00 (−879.55–3743.55)	370.00 (−120.12–860.12)
Asperger is an asset	Promotes that Asperger can be seen as a gift and make you think outside the box	3 (6%)	5277.67 (−16,498.78–27,054.11)	387.67 (−1146.21–1921.54)	137.00 (−384.34–658.34)
Unable to understand the message	Tweets that were hard to understand, decode, or did not make sense	2 (4%)	3688.00 (−40,936.19–48,312.19)	1100.00 (−12,876.83–15,076.83)	182.00 (−2067.00–2431.00)
Asperger is not an excuse	Promotes that one should not use Asperger as an excuse for being rude	1 (2%)	2300.00 (.)	699.00 (.)	96.00 (.)
Informative about Asperger	Neutral and informative content about the Asperger, links to online news or articles	1 (2%)	1300.00 (.)	602.00 (.)	34.00 (.)
Reaching out to a celebrity	Actively making contact with a famous person on behalf of a child with Asperger	1 (2%)	1800.00 (.)	734.00 (.)	19.00 (.)
Total		49 (100%)	7197.92 (4547.54–9848.30)	1477.92 (932.45–1985.00)	375.94 (193.45–558.43)
*p*-value			0.557	0.726	0.214

Kruskal–Wallis test.

## Data Availability

The study data can be obtained from the corresponding author: ingjerd.skafle@hiof.no.
